# Spontaneous Massive Vulvar Edema in Pregnancy: A Case Report

**DOI:** 10.1155/2018/7651254

**Published:** 2018-10-01

**Authors:** Olivier Mulisya, Mbusa Mastaki, Tambavira Gertrude, Kyakimwa Tasi, Jeff K. Mathe

**Affiliations:** ^1^Department of Obstetrics and Gynecology, Femme Engagée pour la Promotion de la Santé Intégrale (FEPSI) Hospital, Butembo, Democratic Republic of the Congo; ^2^Catholic University of Graben, OBS/GYN Department, Democratic Republic of the Congo

## Abstract

Spontaneous massive vulvar edema in pregnancy is unusual and a cause for concern. This condition should be taken seriously since it might be caused by some conditions such as preeclampsia, diabetes, vulvovaginitis, severe anemia, and neoplasms. We report a case of massive vulvar edema in a 15-year-old primigravida following tocolysis therapy at 33 weeks of gestation. Other causes of vulvar edema were excluded. The vulvar edema appeared spontaneously after tocolysis and rapidly increased in size, associated with severe vulvar pains. The vulvar edema resolved progressively with antibiotics, corticoids, and analgesics. The patient delivered by spontaneous vaginal delivery a term live newborn with an unremarkable postpartum period. The aim of this report is to alert clinicians that conservative attempts could be considered for vulvar edema complicating tocolysis.

## 1. Introduction

Spontaneous massive vulvar edema is uncommon during pregnancy with management challenges and significant patient discomfort especially pain and difficulty in ambulation [1]. In literature, the cases of the massive vulvar edema have been described associated with multiple pregnancy, diabetes, hypoproteinemia, preeclampsia, tocolytic therapy, vulvovaginitis, and severe anemia [1–4]. But very rarely is the occurrence after tocolytic therapy.

Vulvar edema has also been reported to result from infections, neoplasms, congenital lymphatic anomalies, trauma, inflammatory, and metabolic disorders [4, 5]. Vulvar edema should also be considered as in important marker for seropositive syphilis in pregnancy as reported in a study among pregnant Mozambican women [6].

In this report we describe a case of spontaneous massive vulvar edema following tocolytic therapy in a 15-year-old primigravida child who presented at 33 weeks of gestation with preterm labor.

## 2. Case Report

A 15-year-old primigravida was admitted at gestational age of 33 weeks and 3 days by ultrasound scan with complaint of intermittent lower abdominal pain with no per vaginal bleeding. Her prenatal care was done regularly in our hospital and the routine prenatal tests (hemoglobin level, syphilis, human immunodeficiency virus serologies, bacteriuria, glucosuria, and proteinuria by urine test strips) were unremarkable and she was of blood group O Rhesus positive. Her blood pressure was in normal range for her three visits. There was no history of urinary tract infection, preeclampsia, or diabetes. There was no reported drug or food allergy. Of note is that her pregnancy was the consequence of rape.

On admission, her general condition was normal, with a height of 143 cm and weight of 52 kgs.

She was afebrile, not pale with no general edema, having no jaundice and her blood pressure was 100/60 mmHg and her pulse rate was 82 bpm. The abdominal examination revealed a gravid uterus with fundal length of 28 cm, longitudinal lie, and cephalic presentation. Fetal heart rate was normal with uterine contractions. On pelvic examination, vulva and vagina were normal. The cervix was firm, posterior, and long admitting a tip of finger. Fingers were stained with whitish discharge. The diagnosis of threatened preterm labor and urinary tract infection was made and the patient was treated by tocolysis therapy with intravenous salbutamol 1,5 mg in 500 ml dextrose 5% at the rate of 7 drops per minute, intravenous ampicillin 1 g two times a day, and intramuscular diclofenac 75 mg two times a day.

Investigations found her hemoglobin at 10.5 g/dl, while on other laboratory examination, urinalysis and vaginal swab were unremarkable.

On day 3, she was improving with no lower abdominal pain and the tocolysis was stopped.

However, on day 4, she complained of vulvar edema. Her blood pressure was 110/80 mmHg, fetal heart rate in normal range with no palpable uterine contractions. On pelvic examination, the vulva was edematous and the diagnosis of vulvitis was suggested. Intravenous gentamycin 80 mg two times a day and oral metronidazole 500 mg three times a day were added on her treatment for 5 days, plus oral ibuprofen 400 mg two times a day as intramuscular diclofenac was already stopped.

**Figure 1:**
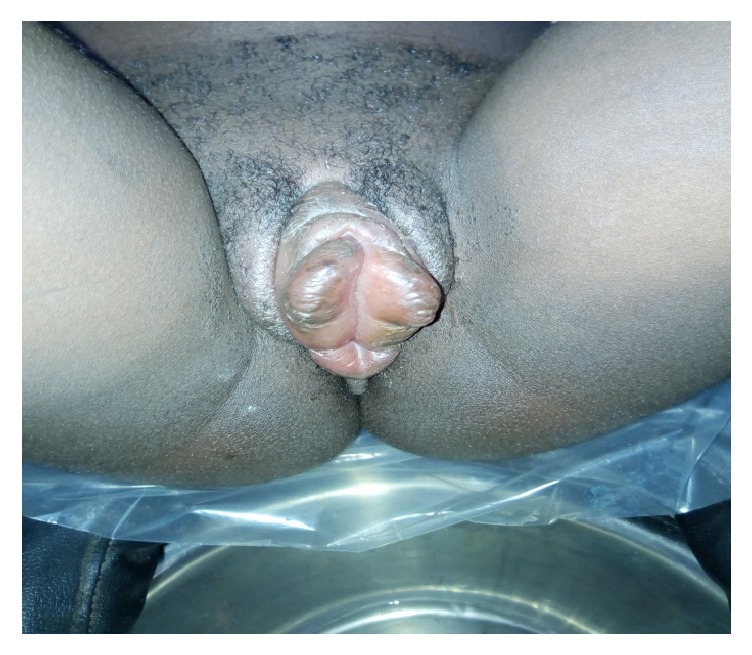
The vulvar edema in day 6.

**Figure 2:**
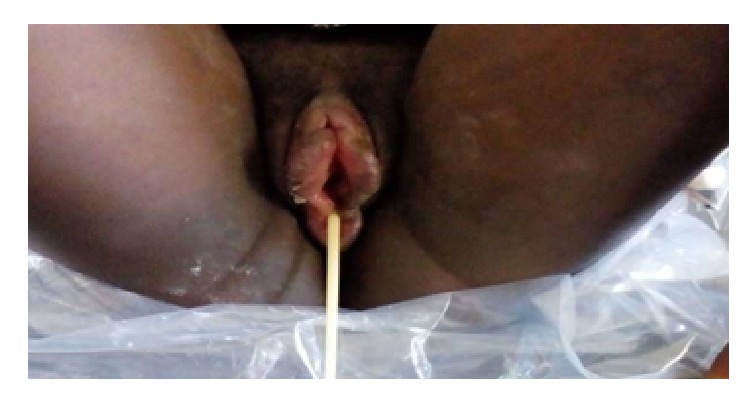
The vulvar edema in day 7, with urinary catheter.

On day 6, the swelling was getting worst despite the treatment and the patient had persistent vulvar pain with difficulty in ambulation and dysuria (Figure 1). Her vital signs were in normal range. Some laboratory tests were requested (the white blood cells were 4,400/mm3, the albuminuria was negative, and the random blood sugar was 82,4 mg/dl) and an obstetrical ultrasound scan revealed a single live, intrauterine fetus at 33 weeks and 6 days. The patient was then treated with intravenous ceftriaxone 1 g two times a day for 7 days, dexamethasone 12 mg once a day for 5 days, and diclofenac suppositories 100 mg two times a day for 3 days as she was in severe pain and ibuprofen was stopped. A urinary catheter was inserted with difficultly due to the edema, to allow easy drainage and the urine output was in the normal range (Figure 2).

**Figure 3:**
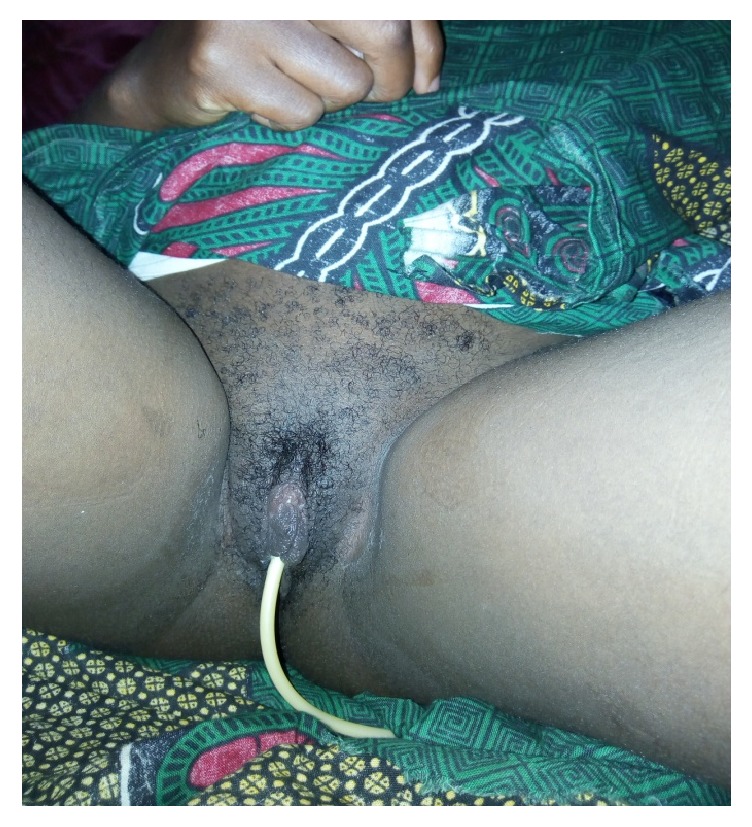
The vulvar edema resolved in day 10.

Under the above treatment, she progressively improved and on day 10, the patient was doing well without swelling of the vulva (Figure 3) and the urinary catheter was removed. She was kept on ward until the onset of labor at term and for close monitoring.

She delivered at 39 weeks and 3 days by spontaneous vaginal delivery a live newborn boy, with weight of 2360 g and Apgar score of 9 at the first minute and 10 at the fifth minute.

## 3. Discussion

In this report we described a case of massive vulvar edema following tocolysis of preterm labor.

The vulvar edema is an extremely rare complication of pregnancy. In literature, cases of the massive vulvar edema have been described associated with multiple pregnancy, diabetes, hypoproteinemia, preeclampsia, tocolytic therapy, vulvovaginitis, and severe anemia [1–4]. But very rarely is the occurrence after tocolytic therapy. For this case, it is still a hypothesis that the edema might have occurred due to the salbutamol therapy. Other studies have shown the same [5, 7, 8]. However, there is a need for further studies to confirm and explain the mechanism behind tocolysis to get vulvar edema.

With edema, one might have thought of hypoproteinaemia as the cause. But this was excluded clinically by the good general condition on admission, with good nutritional status without pedal, lower limb, or generalized edema.

There is no standard way of managing threatened preterm labor. The tocolysis protocol varies from a setting to another and due to the available drugs. Brittain et al. managed their patient with a combination tocolysis with intravenous ritodrine and magnesium sulfate; Trice L et al. used magnesium sulfate, nifedipine, and terbutaline and in a case report by Awwad JT et al., vulvar edema occurred in a twin gestation while on intravenous magnesium sulfate tocolysis. However our patient was managed with only salbutamol.

As for the management, there is no standard way of managing this condition, as there were only few cases reported in the literature [5, 7, 8]. Some manage surgically by incision and drainage or puncturing the edema or by mechanical drainage [9, 10], while others prefer conservative management [11]. Moreover, it is important to find the cause, as management is best influenced by the cause of edema [2]. Some authors report that in most cases, vulvar edema resolves spontaneously after delivery [1]. For this particular case, since patients with vulvar edema might be considered as at high risk, close monitoring was instituted as stipulated by other authors [12], and this could explain the reason that we kept our patient until the delivery. Also, since the diagnosis of threatened preterm labor and urinary tract infection was the first impression diagnosis on admission, the patient was managed by tocolysis (intravenous salbutamol according to our local protocol) and antibiotics (Intravenous ampicillin) as the laboratory tests were going on.

A trial of triple intravenous antibiotics and local skin care proved ineffective in a study by Brittain et al. 1991. But our patient was only managed on the triple antibiotics, corticoids, and analgesics.

Finally, there is no fatality in most reported cases of vulvar edema after tocolysis. Trice L et al., 1996, reported two cases of tocolysis-induced vulvar edema with no fatality and resolved after repositioning the patient in one case and after cesarean section in the other.

## 4. Conclusion

Treatment of vulvar edema is necessary, since it may be alarming to the patient, painful, and uncomfortable and may cause occlusion of the vulva openings. The patient with vulvar edema merits special attention, identification, and treatment of the associated factors is vital to its management.
